# Virtual Reality During Chemotherapy Infusion: An Innovative Intervention in Holistic Nursing Practice

**DOI:** 10.1097/HNP.0000000000000616

**Published:** 2024-06-19

**Authors:** Francesco Burrai, Maria Grazia De Marinis, Michela Piredda

**Affiliations:** **Author Affiliations:** Department of Biomedicine and Prevention, PhD School in Nursing Sciences and Public Health, University of Rome “Tor Vergata,” Rome, Italy (Dr Burrai); Department of Medicine and Surgery, Research Unit Nursing Science, Campus Bio-Medico of Rome University, Rome, Italy (Ms De Marinis); and Department of Medicine and Surgery, Research Unit Nursing Science, Campus Bio-Medico of Rome University, Rome, Italy (Dr Piredda).

**Keywords:** cancer, chemotherapy, Virtual Reality

## Abstract

Patients with cancer receiving infusional chemotherapy show negative symptoms such as worry about their survival, anxiety, anguish, depression, fear, magnified perception of the passage of time, and difficulty managing boredom. Patients also suffer various side effects produced by chemotherapy such as nausea, vomiting, pain, and fatigue, which, together with psychological distress, drastically reduce their quality of life and adherence to therapy with a corresponding reduction in the probability of the individual's survival. Virtual Reality is one of the most innovative and promising digital health interventions, capable of quickly and effectively producing a positive influence on the psychosomatic axis, improving patients' quality of life during chemotherapy. Virtual Reality, through its 3-dimensional multisensory technology, isolates sensory channels from the negative external environment and enables an experience of being physically and psychologically present within virtual scenarios, in which patients can perceive sensations, emotions, cognitions, and interactions as if they really were in different surroundings. This article systematically expounds the scientific conditions necessary for effective, appropriate, and safe implementation of Virtual Reality interventions in holistic nursing practice, describing the underpinning conceptual framework, the types, technological characteristics, methods of use, duration, type of virtual content, and implementation procedure of Virtual Reality.

## INTRODUCTION

When individuals are diagnosed with cancer, their existence is turned upside down. They wonder how long they will survive, and they typically feel helpless, alone, angry, resentful, fearful, and concerned for their family.^1^

To improve survival, most patients must start treatment with intravenous chemotherapy (IC), which, however, can present short-term side effects such as anxiety, pain, fever, diarrhea, nausea, vomiting, gastrointestinal disturbances, and stomatitis, and long-term side effects such as fatigue, alopecia, renal toxicity, and sexual dysfunction, and conditions that decrease quality of life and increase physical and psychosocial distress.^2^

Treatment with IC represents an existential event with strong psychological and social impact, because the IC is infused in hospital health care environments, which present negative visual, auditory, and olfactory stimuli that in their turn can also produce anticipatory nausea and vomiting.^3^ Patients have to leave their home, a familiar and safe environment, to go to an unfamiliar place that can create worry, the fear of side effects, and a state of anxiety due to observing the treatment of other patients besides their own.

A negative factor with a strong potential impact is the duration of each IC session, which can be several hours, depending on various factors such as age, treatment tolerance, therapeutic protocol, and biology and stage of the cancer. The perception of the passage of time is related to an individual's psychological and emotional condition.^4^ During IC, the perception of time is magnified because of the duration of IC, the static position, and the difficulty of managing boredom. This situation focuses attention on unpleasant signals that the body sends during the session, accentuating and complicating the feeling of discomfort and unease during IC. Patients experiencing distress and anxiety tend to perceive time as passing very slowly, overestimating the time that has passed.^4^ All these conditions can produce high levels of stress and reduce adherence to IC and consequently involve a risk of drug dose reduction, administration delay, and IC discontinuation, reducing the efficacy of treatments and thus the probability of survival.^5^

To improve patients' quality of life during IC, it is essential to introduce interventions that can quickly and effectively distract patients from the negative stimuli in which they are immersed, and that are safe and easy to use. Virtual Reality (VR) has all the required characteristics, and today it represents one of the most promising innovative digital health interventions.

### Virtual Reality (VR)

VR is a multisensory environment produced by a technological interface, which allows for an experience of being physically and psychologically present within a virtual environment, in which the patient can perceive sensations, emotions, cognitions, and interactions as if they were actually real.^6^ Using immersive, multisensory, and 3-dimensional technology, VR gets considerably closer to the experiential reality of the individuals, isolating their perceptive channels and immersing them completely in the experience on a sensory level, creating a psychological state in which they experience an illusion of reality. In this way, by isolating the patients from the environment that surrounds them, VR masks the sights and sounds that generate fear.

During IC, the patient's real space-time dimension is transferred to another completely different space-time dimension determined by VR. Although in the IC space-time dimension the patient is a passive subject, who undergoes both the spatial component of the physical environment in which the IC is administered and the relative time for the infusion, in the new space-time dimension created by VR, the patient is an active subject who can choose at any time which virtual place to enter and how long to stay there.

In psychoanalytic terms,^7^ the motivational force that impels the person to choose a particular virtual environment is based on the pleasure principle, in which the contents of VR are chosen because they gratify the person and produce pleasure, satisfaction, and well-being. Therefore, the patients immersed in VR have sought “another" experience, in which they are no longer physically and psychologically in the IC experience, because VR has diverted their attention from unpleasant stimuli toward pleasant and interesting stimuli, replacing a negative experience with a better one.

In terms of neuroscience integrated with psychoanalysis, VR could stimulate the search not only for pleasure but also for the avoidance of pain, important motivational elements, and fundamental variables as regards survival.^8^ In terms of positive psychology, VR could positively influence the person's mood during IC by decreasing negative emotions and increasing the intensity and frequency of positive emotions that stimulate inner resources and improve quality of life.^9^

As stated previously, VR alters the perception of time. Patients who were treated with VR during IC showed that their temporal perception was different: time was perceived as passing more quickly.^10^ The increase in the speed of the passing of time could be due to the distraction produced by VR, and in terms of the simulation-accumulation cognitive model,^11^ by moving the focus of attention from negative stimuli toward virtual scenes, VR has also diverted attention from the processing of temporal information.

VR can considerably improve patients' ability to tolerate and adhere to IC, which is critical for their survival. In fact, patients who knew that they would be able to use VR during IC felt encouraged to keep the IC appointment.^10^

#### VR types

VR can be classified according to the depth of the multisensory experience into immersive VR (iVR), semi-immersive VR (sVR), and nonimmersive VR (nVR), while according to the level of interactivity, it can be classified into contemplative VR (cVR) and participatory VR (pVR). iVR uses technological equipment consisting of a head-mounted display (HMD) headset and tactile and movement devices such as haptic gloves and sensory trackers. This type of VR has the ability to completely isolate patients from their real environment during IC, immersing them in the virtual experience in a multisensory way.

In sVR, the patients use a viewer that allows them to watch 3-dimensional content that is projected onto walls through surround rear-projection devices and screens that reproduce stereoscopic images from a computer. The patients are not completely isolated from the environment in which they are receiving IC. nVR does not use viewers or a dedicated HMD system and the virtual content is accessed by looking at a monitor connected to a computer. cVR enables patients to immerse themselves in the virtual content without any sensorimotor interaction with it. Patients “passively” observe the scenes with varying degrees of immersion and are able to choose between the contents, or perform tasks communicated by the scenes, such as relaxation exercises, awareness of their sensations related to well-being, or states of meditation, but they cannot manipulate or control the virtual environment. In pVR, the patient becomes the protagonist within the virtual world, interacting with it in a sensorimotor way, for example, the patient can draw, play with, and modify objects.

#### Technology

Technology is the fundamental variable for transferring patients' experience from the physical place where they are undergoing IC to a nonphysical place, where they can perceive their experience as real and consequently be deeply present in an experience far removed from suffering. The most advanced technologies that guarantee this type of experience are those of iVR using the latest-generation technology. The equipment consists of an HMD headset, with high-power onboard processors, a viewer made up of full high-definition and liquid crystal display lenses with high pixel density, with stereoscopic vision combined with various sensors for eye and face tracking, ensuring clear, precise, and deep vision with high-resolution, vivid colors, and 360° movement without distortion. At the audio level, spatial audio-processing technology is used, which allows the perception of high-definition, clear, natural sound, with high dynamic range. The sound can be heard both through the built-in speakers and through earphones. At a tactile level, the haptic gloves or sense gloves allow patients to feel the size, stiffness, and resistance of virtual objects, and to hold, push, and touch them. These technologies are comfortable and low-weight. In the few studies that have used iVR during IC, the VR equipment did not have haptic gloves or sense gloves,^12^-^15^ reducing the technologies' ability to produce a deep immersion experience and reducing their effectiveness in improving the quality of life of patients during IC.

#### Length

There is no consensus on the optimum duration of immersion in VR to produce effective results during IC. VR immersion length can range from 15 minutes for iVR^13^ to several hours for nVR.^16^ VR immersion length is connected to safety to avoid the possible adverse effects of prolonged use.^17^ These adverse effects are described as cyber sickness and must always be assessed with validated tools such as the Virtual Reality Symptom Questionnaire.^17^

When VR is proposed to patients receiving IC, it could create a surprise effect and a strong attraction to this intervention, conditions that could represent confounding variables for the measurement of the emotional states produced by virtual environments.^12^ To reduce this risk, a phase called “familiarization” is used, where, before starting the real treatment with VR, the patient gets to know the technology and some scenarios gradually, guided by the health providers,^12^ and practices navigating among virtual scenes.^18^ This phase can take place 10 minutes before the start of the real immersion on the first day of VR use^18^ and 5 to 10 minutes before the start of the real immersion on subsequent days.^12^

#### Scenarios

The virtual scenarios most used during IC are the natural environment.^12^-^15^ Nature scenes, with their own colors, environments, and sounds, have a strong distractive capacity.^19^ They produce a psychophysical state characterized by reduction of negative emotions and stress and increased positive emotions, relaxation, calm, and inner peace, bringing to mind positive memories that decrease anxiety and depression.^20^

## CONCEPTUAL FRAMEWORK

The conceptual framework is based on a unitary, integrated psychological and neurological model.^21^ The VR technology system consists of an HMD headset and haptic gloves and sensory trackers. On the visual level, the HMD system guarantees stereoscopic vision allowing the perception of the 3-dimensionality of virtual objects. On the auditory level, it provides high-definition stereophonic sound that allows complete audio immersion in the virtual environment. Finally, on the tactile level, it sends signals such as vibration, weight, temperature, and shape that allow users to manipulate virtual objects as if they were physically present. The 3 sensory outputs are sent simultaneously by the hardware and software system to the relative visual, auditory, and somatosensory systems, and they reach the brain through the nerve pathways to be processed by neuronal recoding. In neuronal recoding, the state of perception of the patient's body in space-time is altered by the VR contents. The perception of the body occurs both through the cerebral cortex with activation of consciousness^22^ and through the autonomic nervous system, defining the perception of possessing “a body” and its space-time location.^23^ In predictive coding theory terms,^24^ the brain creates 3 types of simulation experience during VR: (1) sensory and motor experiences; (2) experiences related to the subject's expectations^25^; and (3) experiences of actions to face imminent events.^26^ VR affects the limbic and paralimbic cortices, which provide cortical control of the body's internal environment, and they send visceromotor predictions to the hypothalamus and brainstem nuclei, which regulate autonomic, neuroendocrine, and immune responses.^26^

Several brain structures are activated during VR: (1) the frontal lobe is involved in motor control and working memory^27^; (2) the temporal lobe is activated in the processing of episodic memory^28^; (3) the mean temporal gyrus is implicated in sound recognition^29^; (4) the parietal lobe is involved in creating the sense of presence^30^; (5) the superior parietal lobe is activated in the mental imaging of the body in space^31^; and (6) the occipital lobe is involved in the creation of visual images.^32^ Importantly, VR could activate mirror neuron action and neuroplasticity, influencing observational learning and promoting imitation.^33^ At the level of the somatosensory system, 3 essential functions are activated by VR: (1) exteroceptive: perception of stimuli coming from outside the body; (2) interoceptive: perception of stimuli from inside the body; and (3) proprioceptive: with the function of perception and control of body balance.^34^ The global system of neuronal recoding thus produces perception by the consciousness of images, sounds, and tactile sensations, or rather the virtual scenarios produced by VR.

On the psychological level, the focus of attention is shifted toward the contents of the virtual scenarios, and the degree of shifting of attention, or rather of the distraction, is directly proportional to the patient's sense of presence in the natural environment scenarios, of the interest that the patient takes in the scenarios, the patient's perception of the pleasantness of the environment, and the patient's interactivity with the virtual environment. Interactivity is the fundamental aspect that characterizes iVR; it is that process of perception in which the patient can interact with the virtual context, providing a high level of involvement and realism.^35^ Interactivity creates a strong sense of presence, a subjective psychological state of “being there,” in the virtual world, without any perception of technological mediation.^14^

To maintain the initial involvement in the new immersive virtual environment, it is necessary to allow continuous interaction with this virtual space, to be able to first explore it visually as is done in any new real environment, and then to physically interact with it.^35^ The use of tactile devices makes it possible to effect the interactions and congruent responses of the patient.^36^

Shifting the focus of attention by means of VR produces a state of distraction, allowing patients' attention to be diverted away from unpleasant emotions and directed toward positive stimuli to reduce unpleasant moods.^10^ At this level, the greater the degree of shift of attention toward the contents of the scenarios, the greater will be the level of distraction from negative hospital stimuli and also the depth of the sense of presence and the patient's perception of being truly “there,” as an active participant in the virtual scenario.

Sense of presence can be defined as “the subjective experience of being in a place or environment, even when physically located elsewhere,”^37^ and consequently, people respond by behaving in a manner consistent with the experience perceived.^38^ The state of presence is the subjective sense of the reality of the self in the world and of the surrounding world as it is perceived,^39^ and the effectiveness of virtual environments is closely connected to the depth of the sense of presence perceived by people. The subjective sense of presence can present 2 psychological conditions: “Place Illusion” and “Plausibility.”^38^ “Place Illusion” is the illusion of perceiving a place created by VR as real, while “Plausibility” is the illusion that the events experienced in VR are actually happening, with the knowledge that they are not happening in physical reality. When “Place Illusion” and “Plausibility” are both present in consciousness, people respond to VR realistically according to what they see, hear, and touch.^40^ People with the ability or tendency to be easily involved or immersed in a situation may benefit more rapidly from distraction interventions such as those produced by VR.^37^ At this level, there is therefore a sequential bidirectional influence between attention, distraction, and presence, which are connected in a directly proportional way, that is, as the depth of activation of one of the cognitive components increases or decreases, it will immediately influence the other connected cognitive mechanisms.

Activation by the virtual scenarios of the cognitive mechanisms of attention, distraction, and presence also influences the time variable, the perception of the passage of time defined as temporality, influencing the links between treatment time, VR time, and negative thoughts, emotions, and sensations. The 3 cognitive mechanisms influence the perception of the passage of time in a directly proportional way, that is, as activation by virtual scenarios of depth of attention, distraction, and presence increases or decreases, the greater or lesser will be the increase in speed of the passing of time as perceived by the patient.

The sequential connection between VR HMD headset, VR scenarios, attention, distraction, presence, and temporality forms the complex structure of the immersive experience, which produces a convincing illusion of being in a real world.^41^ In a condition in which VR were able to perfectly simulate all the senses, the person would be immersed in a state that can be defined as superrealism, in which it was impossible for the person to perceive actual reality.^40^ High levels of interaction^42^ and the level of immersion greatly influence the person's experience during VR. A VR session that produces deep immersion creates a strong sense of presence in relation to virtual contents, greatly influencing people's ability to be distracted and to refocus their attention,^41^ preventing patients from focusing on competing negative stimuli.^43^

The immersive experience contains the whole complex of interactive information between VR and the patient's psychosomatic response, producing effects at the level of cognitive, emotional, and physical outcomes. A positive cognitive outcome response, represented as increased control, tolerance, or reduction of stress, can be explained in terms of the stress and adaptation model of Lazarus and Folkman.^44^ In this model, VR stimulates new cognitive skills by becoming a coping technique.

On an emotional level, a reduction in the level of anxiety, in the number and intensity of negative emotions, could be explained by the positive psychology of Fredrickson.^45^ By shifting attention toward a virtual environment perceived as positive, VR produces a reduction of negative emotions; consequently, patients have useful new emotional resources available, enabling them to deal better with negative situations such as IC.^46^

Considering the cognitive and emotional levels as a whole, VR could be used as a systematic desensitization intervention, entailing an antagonistic response, for example, to the anxiety that patients experience during IC, in order to condition and weaken the link between anxiety-provoking stimuli and anxiety reaction.^47^

At the level of the physical component, the immersive experience influences the hippocampus and amygdala, which play an important role in the formation of positive and negative emotional states such as stress, anxiety, fear, and depression. The immersive experience reduces the activation of the hypothalamic-pituitary-adrenal axis, with a consequent reduction in the perception of stress, fear, and anxiety.

The global improvement of cognitive, emotional, and physical states can be explained using 2 theories such as the Stress Reduction Theory^48^ and the attention restoration theory.^49^ In these models, the immersive experience that produces well-being is produced by the use of virtual scenes of natural environments, capable of effectively and quickly shifting attention from negative stimuli to an environment free from stressful stimuli. The Figure illustrates the conceptual psychoneurological framework underpinning the effect of VR.

**FIGURE.**
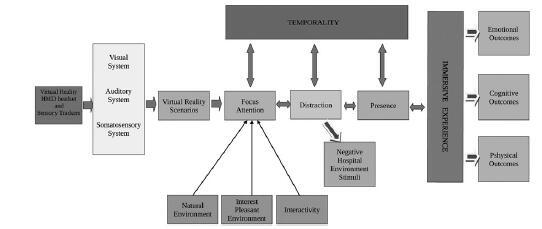
The conceptual psychoneurological framework of Virtual Reality. HMD indicates head-mounted display.

## IMPLICATIONS FOR NURSING PRACTICE AND FURTHER RESEARCH

VR is a digital health intervention, which, in order to be implemented effectively, appropriately, and safely in nursing care for patients undergoing IC, must have the following characteristics: (1) technology consisting of an HMD headset with haptic gloves; (2) pVR; (3) iVR; (4) virtual scenarios mainly consisting of natural environments; (5) length of the immersion in the virtual scenarios of a maximum of 30 minutes, followed by a 15-minute break, to reduce the risk of cyber sickness; (6) 10-minute familiarization phase on the first day of VR use; and (7) assessment of potential cyber sickness after each use of VR with the Virtual Reality Symptom Questionnaire.

An important aspect is the funding of specific programs for the study of the effects of VR. For example, in Italy there are not specific grants for VR from funding agencies in the public, commercial, or not-for-profit sectors.

Further studies should focus on the following research aims: (*a*) to investigate which immersion time in virtual environments produces the best effects on the psychosomatic axis; (*b*) to find the best virtual contents customized to the physical, psychological, cultural, social, personal, and spiritual aspects of the patient; (*c*) to study any cyber sickness produced by VR; and (*d*) to study levels of patient satisfaction with VR.

## CONCLUSIONS

The latest-generation iVR, thanks to the increase in computing power, the use of more sophisticated software, better optical components, low latency (the delay between action and reaction), and wide field of view and increased interactivity, has significantly improved the VR experience, becoming a powerful distractive intervention for patients during IC. However, compared with the millions of patients who are treated with IC every day and suffer both psychologically and from its side effects, VR is still scarcely used by nurses. VR represents a modern intervention that can be implemented in holistic nursing practice because it is effective, safe, easy to use, has low cost, and is appreciated by patients. It is essential to train nurses in the use of VR and to build teams of nurses, oncologists, information and technology specialists, and patients who can codesign and coproduce specific virtual scenarios.^50^ VR is a digital health intervention with the potential to play an increasingly decisive role in improving patients' quality of life in oncology and in many other care fields.
